# The SINE Compound KPT-350 Blocks Dystrophic Pathologies in DMD Zebrafish and Mice

**DOI:** 10.1016/j.ymthe.2019.08.016

**Published:** 2019-09-03

**Authors:** Rylie M. Hightower, Andrea L. Reid, Devin E. Gibbs, Yimin Wang, Jeffrey J. Widrick, Louis M. Kunkel, Jenna M. Kastenschmidt, S. Armando Villalta, Thomas van Groen, Hua Chang, Savanna Gornisiewicz, Yosef Landesman, Sharon Tamir, Matthew S. Alexander

**Affiliations:** 1 Department of Pediatrics, Division of Neurology, University of Alabama at Birmingham and Children’s of Alabama, Birmingham, AL 35294, USA; 2 UAB Center for Exercise Medicine (UCEM), Birmingham, AL 35294, USA; 3 Division of Genetics and Genomics at Boston Children’s Hospital, Boston, MA 02115, USA; 4 Department of Genetics at Harvard Medical School, Boston, MA 02115, USA; 5 Harvard Stem Cell Institute, Cambridge, MA 02138, USA; 6 The Manton Center for Orphan Disease Research at Boston Children’s Hospital, Boston, MA 02115, USA; 7 Department of Physiology and Biophysics, University of California-Irvine, Irvine, CA 92697, USA; 8 Institute for Immunology, University of California-Irvine, Irvine, CA 92697, USA; 9 Department of Cell, Developmental, and Integrative Biology, University of Alabama at Birmingham, Birmingham, AL 35294, USA; 10 Karyopharm Therapeutics, Newton, MA 02459, USA; 11 Department of Genetics at the University of Alabama at Birmingham, Birmingham, AL 35294, USA; 12 Civitan International Research Center at the University of Alabama at Birmingham, Birmingham, AL 35294, USA

**Keywords:** KPT-350, SINE, XPO1, CRM1, DMD, muscle zebrafish, muscle inflammation

## Abstract

Duchenne muscular dystrophy (DMD) is an X-linked muscle wasting disease that is caused by the loss of functional dystrophin protein in cardiac and skeletal muscles. DMD patient muscles become weakened, leading to eventual myofiber breakdown and replacement with fibrotic and adipose tissues. Inflammation drives the pathogenic processes through releasing inflammatory cytokines and other factors that promote skeletal muscle degeneration and contributing to the loss of motor function. Selective inhibitors of nuclear export (SINEs) are a class of compounds that function by inhibiting the nuclear export protein exportin 1 (XPO1). The XPO1 protein is an important regulator of key inflammatory and neurological factors that drive inflammation and neurotoxicity in various neurological and neuromuscular diseases. Here, we demonstrate that SINE compound KPT-350 can ameliorate dystrophic-associated pathologies in the muscles of DMD models of zebrafish and mice. Thus, SINE compounds are a promising novel strategy for blocking dystrophic symptoms and could be used in combinatorial treatments for DMD.

## Teaser

Hightower et al. demonstrate the therapeutic use of a SINE (selective inhibitor of nuclear export) compound, KPT-350, to treat Duchenne muscular dystrophy (DMD) pathologies in two vertebrate animal models. The authors demonstrate these effects are due to an increase in a pro-regenerative macrophage population in dystrophic skeletal muscles. KPT-350 is in clinical trials for other neuromuscular disorders and may hold promise to ameliorate DMD symptoms.

## Introduction

Duchenne muscular dystrophy (DMD) is a progressive neuromuscular disease caused by mutations in the *DYSTROPHIN* gene resulting in the lack of production of functional dystrophin protein.^1^, ^2^ DMD affects approximately 1:5,000 live male births worldwide, making it the most common childhood form of muscular dystrophy. Patients with DMD gradually develop muscle weakness, postural instability, cardiac arrhythmias, respiratory weakness, and loss of ambulation after the first decade of life. The myofiber damage attributed to membrane instability also causes chronic inflammatory responses in dystrophic muscle.^3^, ^4^, ^5^, ^6^ This inflammatory response is characterized by the infiltration of immune cells that produce inflammatory and fibrotic factors that contribute to the progression of DMD.^7^ Isolated DMD patient muscle cells have been shown to express higher levels of collagen and extracellular matrix (ECM) factors compared with heathy muscle cells.^8^, ^9^, ^10^ This progressive increase in endomysial fibrosis is significantly correlated with poor motor outcome and loss of ambulation in DMD patients.^11^ There is no cure for DMD, and corticosteroids are the current primary standard-of-care treatment.^12^, ^13^ The functional preservation seen in patients in response to corticosteroid therapy is thought to be a result of their immunosuppressive properties, which reduces the detrimental fibrotic pathology associated with dystrophin deficiency.^14^, ^15^, ^16^ Anti-fibrotic and anti-inflammatory compounds or biologics that target key drivers of inflammation in DMD, such as interleukin-6 (IL-6), transforming growth factor β (TGF-β), tumor necrosis factor alpha (TNF-α), the nuclear factor κB (NF-κB) signaling pathways, or regulatory T cells, have shown therapeutic efficacy in reducing dystrophic symptoms in dystrophin-deficient mice.^17^, ^18^ Regulatory T cells have been shown to block and/or ameliorate dystrophic symptoms in mouse and canine DMD models.^7^, ^17^, ^19^, ^20^, ^21^, ^22^, ^23^, ^24^, ^25^, ^26^

The nuclear pore functions as a key regulator of intracellular molecules such as proteins, RNA molecules, and ions.^27^, ^28^ The nuclear pore consists of various regulatory proteins called nucleoporins that together form the nuclear pore complex (NPC).^28^ Many of these nucleoporins have direct roles in regulating the transport of key proteins and RNA macromolecules from the nucleus and the cytoplasm. The NPC is an important regulator of key protein cargos involved in cellular differentiation, immune response, apoptosis, and overall transcriptional and translational machinery.^29^ The nuclear protein exportin 1 (XPO1; also called CRM1) has an essential role in protein trafficking that functions as a nucleocytoplasmic regulator of key transcription factors.^30^, ^31^ Recently, direct pharmacological inhibition of XPO1 has been shown to be effective in several types of cancers.^32^, ^33^, ^34^, ^35^ XPO1 also has been demonstrated to interact with polyglutamine (polyQ) proteins that are produced from expanded nucleotide repeats in disorders such as Huntington’s.^36^ Some amyotrophic lateral sclerosis (ALS) and frontotemporal dementia (FTD) patients also have expansion repeats in the *ATXN2* gene that result in the production of polyQ proteins.^37^, ^38^ They have been shown to exhibit defective nuclear export activity, likely regulated by XPO1 function.^39^, ^40^, ^41^, ^42^ A series of pharmacological inhibitors of XPO1 nuclear export function have been developed initially for cancer therapies.^43^ However, recent findings have demonstrated that neurological and neuromuscular disorders containing pathogenic expansion repeats affect XPO1 activity; thus XPO1 could serve as a pharmacological target.^44^, ^45^

KPT-350 is a selective inhibitor of nuclear export (SINE), one member of a series of compounds originally designed by Karyopharm Therapeutics (recently acquired by Biogen and renamed BIIB100) to inhibit XPO1 nuclear export function. Recently, KPT-350 has been shown to block Huntington’s disease pathologies via correcting nucleocytoplasmic transport and preventing mutant HTT protein from aggregating around the NPC.^44^ KPT-350 and other SINE compounds block inflammation and neurotoxicity via the regulation of key transcription factors shown to drive these processes such as NF-κB, inhibitor of κB (IκB), FOXO1, FOXP1, and STAT1. Based on these anti-fibrotic and anti-inflammatory effects, we postulated that treatment with KPT-350 might have a beneficial effect in preventing dystrophic muscle pathology in DMD zebrafish and mice by blocking fibrosis and inflammation in their skeletal muscles. We treated dystrophic zebrafish in short- and long-term experiments via immersion of DMD larvae in KPT-350 (or vehicle control) and evaluated their outcomes. In addition, we performed an expanded evaluation of KPT-350 in the DBA2J-*mdx* (D2*-mdx*) DMD mouse model with an emphasis on skeletal muscle histology, serum biomarkers, and overall functional outcomes. These studies were conducted for the pre-clinical evaluation of KPT-350 in two relevant vertebrate DMD models for overall therapeutic efficacy in blocking dystrophic symptoms.

## Results

### KPT-350 Ameliorates Dystrophic Pathologies and Extends the Lifespan of Dystrophin Mutant Zebrafish

**Figure 1:**
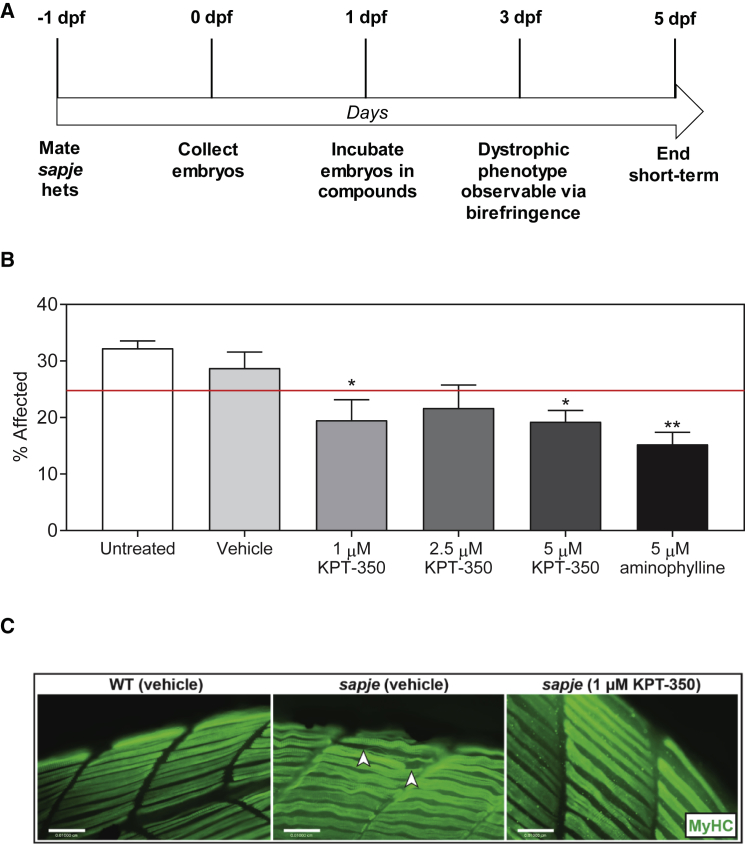
KPT-350 Short-Term Dosing Reduces Percentage of Affected DMD Zebrafish and Improves Overall Myofiber Histology (A) Schematic for short-term KPT-350 treatment. (B) Summary graph of total percent of affected embryos from paired sapje heterozygous matings after vehicle treatment, KPT-350 treatment (0.1 μM, 1 μM, 5 μM), or 5 μM aminophylline (positive control) (n = 20 fish per cohort; experiments were repeated three times independently). The red line demarcates 25% expected Mendelian ratio of affected *sapje* homozygote fish. (C) Immunofluorescent staining of fast myosin heavy chain in WT + vehicle, *sapje* + vehicle, and *sapje* + 1 μM KPT-350 treatment. White arrows designate disrupted muscle fiber. The SEM is shown for the averaged experimental results. For each experiment, n = 20 fish per cohort were used, and the experiment was repeated four times independently in a double-blinded fashion.

*Sapje* zebrafish mutants are a well-established model for drug screening and evaluation of muscle pathologies. The *sapje dmd*^*ta222a*^ mutant zebrafish have myofiber detachment from the sarcolemmal membrane, impaired muscle force production, reduced motility, a decreased lifespan with 95% death by 10 days post fertilization (dpf), and an overall pathology that closely resembles the human disease. Zebrafish are also a powerful model for identifying corrective neuromuscular compounds due to their ability to rapidly uptake small molecules through their gills and skin. Dorsal skeletal muscle birefringence, which assesses muscle fiber integrity via polarized light, is a useful tool for analyzing muscle pathologies as observed in *sapje* homozygous mutants and for rapidly evaluating the muscle quality of early (4–7 dpf) zebrafish larvae. To test whether KPT-350 might block or ameliorate dystrophic symptoms, we first performed short-term testing in the *sapje* mutant zebrafish. We mated *sapje* heterozygotes and immersed 1 dpf embryos obtained from timed matings in fish water containing either vehicle (0.01% DMSO/fish water), KPT-350 (1, 2.5, or 5 μM), or 5 μM aminophylline for 4 consecutive days while changing the water every other day in a double-blinded (genotype and compound) fashion (Figure 1A). At 5 dpf, the zebrafish larvae were evaluated for muscle birefringence, and we found a reduction in the overall number of the percent affected fish from the expected 25% Mendelian ratio in the KPT-350-treated cohorts, but not in the untreated or vehicle controls (Figure 1B). We also observed a decrease in the percentage of affected fish in the 5 μM aminophylline-treated positive control cohort similar to the ratios of what we previously observed.^46^ Evaluation of dorsal muscle birefringence revealed that 1 μM KPT-350-treated fish showed no quantifiable muscle wasting phenotype (Figure 1C). Genotyping of the cohorts revealed that drug-treated cohorts had more *sapje* mutant fish compared with vehicle control. These data suggest that both 1 and 2.5 μM doses of KPT-350 were sufficient to block the dystrophic phenotype in the *sapje* mutant fish in the early stages of dystrophic muscle pathologies.

**Figure 2:**
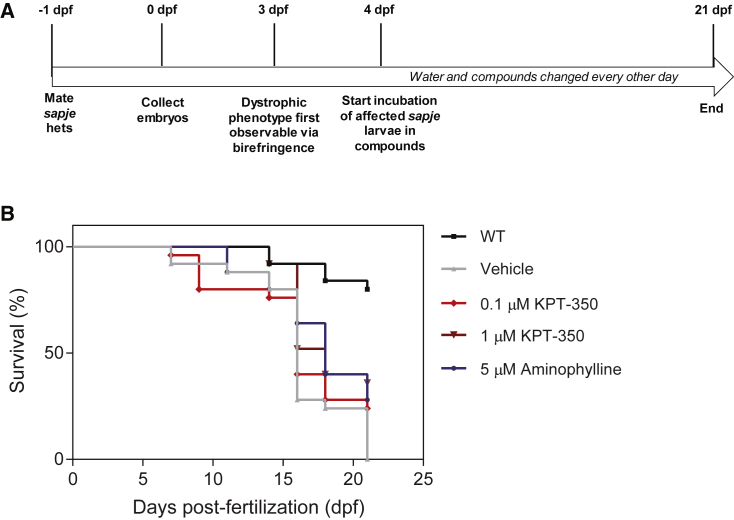
KPT-350 Long-Term Treatment Extends the Lifespan in DMD Zebrafish (A) Schematic for long-term KPT-350 treatment. (B) Survival plot of long-term experiment for WT (untreated), *sapje* mutant vehicle (0.01% DMSO/fish water), *sapje* mutant 0.1 and 1.0 μM KPT-350-treated, and 5 μM aminophylline (positive control)-treated cohorts from 4 to 21 dpf. 1 μM KPT-350 treatment significantly extended lifespan in *sapje* homozygote mutants compared with vehicle treatment. For each experiment, n = 20 fish per cohort were used, and the experiment was repeated three times independently in a double-blinded fashion. Log rank test was used to determine statistical significance.

We next tested whether a long-term KPT-350 treatment drug testing regimen (from 4 to 21 dpf) could minimize dystrophic dorsal muscle pathology (Figure 2A). We used birefringence to screen 4 dpf *sapje* larvae for homozygote mutants (poor muscle fiber integrity) and immersed mutants in water containing either vehicle, KPT-350 (0.1 or 1 μM), or 5 μM aminophylline, previously shown to extend *sapje* mutant survival, as a positive control.^46^ Unaffected wild-type (WT) were assessed as a frame of reference for survival. We observed a significant extension of lifespan with 1 μM KPT-350 treatment (median = 18 days) compared with vehicle treatment (median = 16 days; log rank test) in affected *sapje* homozygote mutants (Figure 2B). These findings in *sapje* mutant zebrafish indicate that KPT-350 treatment may both block and ameliorate dystrophic symptoms and pathologies in a disease-relevant vertebrate model.

### Oral Delivery of KPT-350 Blocks Dystrophic Skeletal Muscle Degeneration and Fibrotic Pathologies in *D2-mdx* Mice

**Figure 3:**
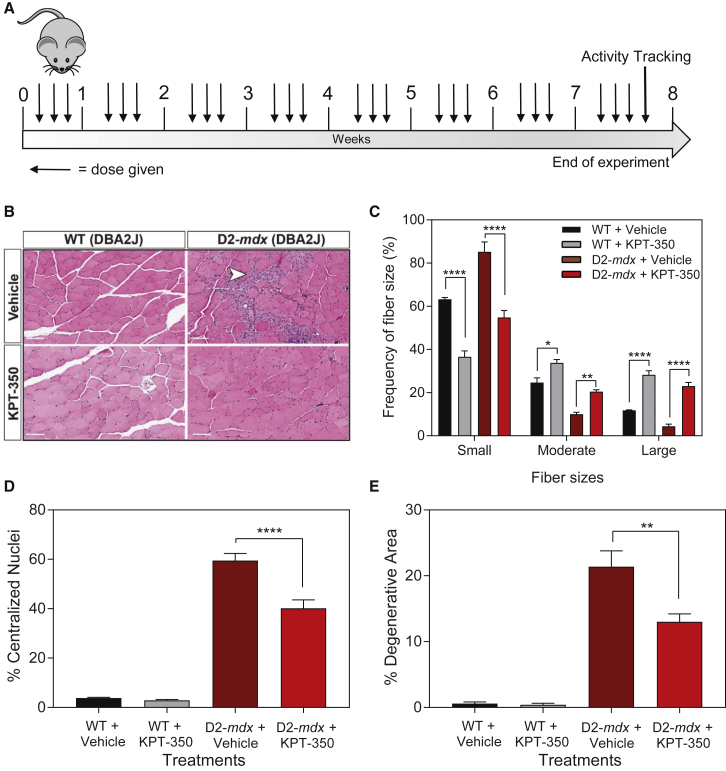
KPT-350 Treatment Improves Histological Hallmarks of Dystrophic Pathology (A) WT and D2-*mdx* mice were given oral KPT-350 at 5 mg/kg three times a week for 8 weeks beginning at 8 weeks of age. (B) Tibialis anterior muscles were sectioned and stained in H&E (representative images shown, original magnification ×20, scale bars: 100 μm). KPT-350 treatment significantly increased the frequency of large-size fibers with a concomitant decrease in small-size fibers in both WT and D2-*mdx* cohorts (C). In addition, KPT-350-treated D2-*mdx* mice had significantly fewer centralized myonuclei (D) and less inflammation (E) than the vehicle-treated D2-*mdx* mice. Mean ± SEM. n = 5. *p < 0.05, **p < 0.01, ***p < 0.001, ****p < 0.0001, two-way ANOVA.

We next pursued expansion of our zebrafish KPT-350 findings in a mouse DMD model. The *mdx* (*DBA/2J*) strain, referred to here as D2-*mdx*, contains the mouse dystrophin exon 23 C>T transition, resulting in a loss of the Dp427m dystrophin protein isoform.^47^, ^48^ This strain exhibits a more severe skeletal muscle phenotype when directly compared with the more commonly used *C57BL6/10ScSnJ mdx* strain, thus making it a more physiologically relevant DMD model for pre-clinical therapeutic evaluation.^49^, ^50^ We evaluated the therapeutic ability of oral KPT-350 given to D2-*mdx* and control WT (*DBA/2J*) mice in the form of peanut butter pellets to ameliorate the dystrophic pathology and improve muscle function. WT and D2-*mdx* mice were treated with either vehicle (0.6% Plasdone PVP K-29/32, 0.6% Poloxamer Pluronic F-68) or KPT-350 (5 mg/kg body weight) three times a week for 8 weeks beginning at 8 weeks of age (Figure 3A). Immediately following euthanasia of animals, muscles were extracted and used for histological and molecular analyses. Histological analysis revealed that KPT-350 treatment significantly reduced dystrophic pathology in D2-*mdx* muscle. First, cross-sectional area (CSA) of the tibialis anterior (TA) was measured, as well as the frequency of either small (0–999 μm^2^), moderate (1,000–1,799 μm^2^), or large fibers (>1,800 μm^2^) in all cohorts (Figures 3B and 3C). In both WT and D2-*mdx* strains, KPT-350 treatment significantly increased the frequency of large-size fibers with a concomitant decrease in small-size fibers (Figure 3C). This suggests that KPT-350 treatment may be preventing muscle atrophy or degradation in D2-*mdx* muscle. In addition, KPT-350-treated D2-*mdx* muscle had significantly fewer centralized myonuclei and smaller degenerative lesions compared with vehicle-treated D2-*mdx* mice controls (Figures 3D and 3E). Taken together, these data indicate that KPT-350 treatment improves the overall dystrophic phenotype because these treated D2-*mdx* mice presented significantly less muscle atrophy, centralized myonuclei, and overall degeneration, all hallmark characteristics of muscular dystrophy.

### KPT-350 Treatment Improves Overall Locomotion and Overall Activity in D2-*mdx* Mice

**Figure 4:**
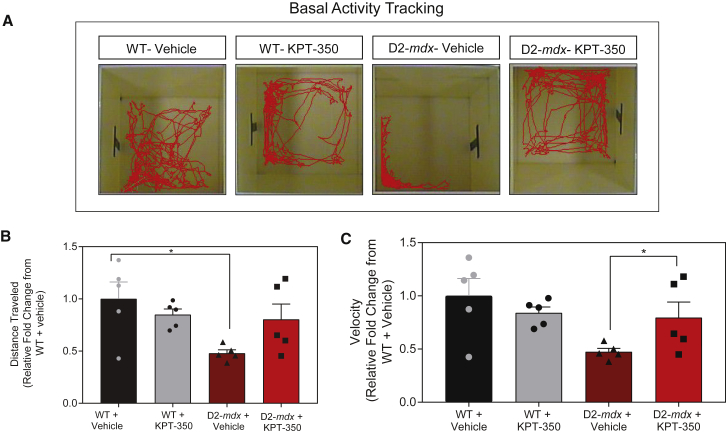
KPT-350 Treatment Improves Overall Locomotion and Overall Activity in D2-*mdx* Mice (A) Representative locomotor activity tracings from WT and D2-*mdx* mice treated with KPT-350 or vehicle. Red tracings designate route of physical movement for each mouse taken 1–2 days prior to the experimental end (14-week-old mice). (B) Summary graph demonstrating quantified locomotor activity tracings of distance traveled by each WT and D2-*mdx* mouse treated with KPT-350 or vehicle. (C) Summary graph demonstrating quantified locomotor velocity of WT and D2-*mdx* mice treated with KPT-350 or vehicle.

Open field activity tracking is a validated way of assessing basal ambulatory function in mouse models of skeletal muscle disease.^51^, ^52^, ^53^ Preservation of ambulation is an important component of therapeutic treatment in patients with DMD; therefore, assessing locomotor activity in D2-*mdx* mice can provide insight into preservation of physical function after treatment. In order to determine the functional significance of short-term KPT-350 treatment, we assessed overall locomotor activity and movement velocity in both drug-treated and vehicle-treated D2-*mdx* mice. KPT-350-treated D2-*mdx* mice demonstrated no significant difference in total distance traveled from vehicle-treated D2-*mdx* mice (Figures 4A and 4B). However, KPT-350-treated D2-*mdx* mice showed a significant increase in overall movement velocity compared with vehicle-treated D2-*mdx* mice (Figure 4C). This demonstrates that KPT-350 prevents rapid decline of overall locomotor activity, and that KPT-350 treatment results in increased locomotor velocity in D2-*mdx* mice.

### KPT-350 Inhibits Inflammatory Cytokines and Improves DMD Serum Biomarkers in D2-*mdx* Mice

**Figure 5:**
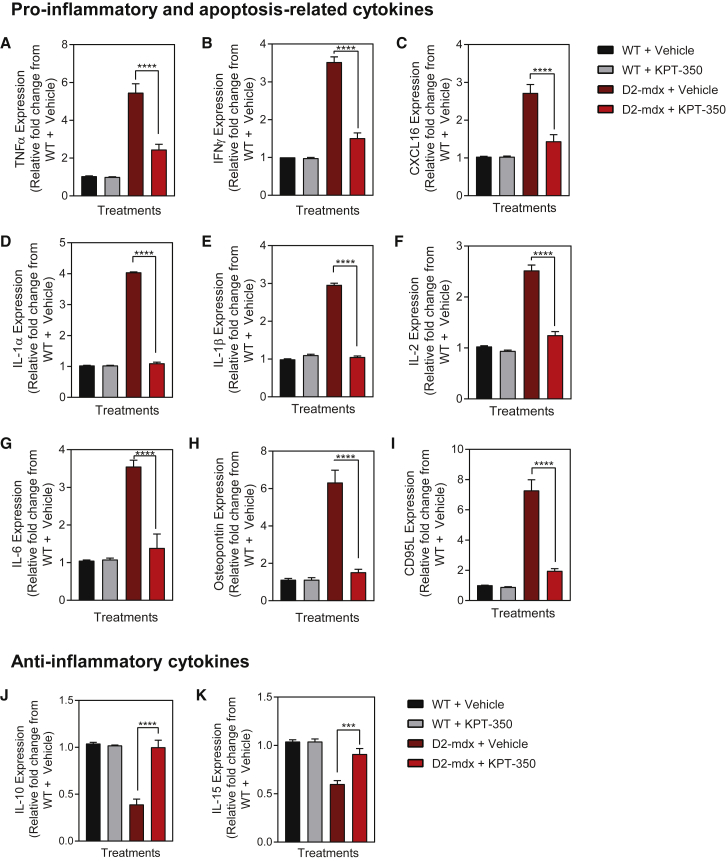
KPT-350 Treatment Results in Decreased Inflammatory Cytokine Expression in D2-*mdx* Mice (A–I) WT and D2-*mdx* mice were given oral KPT-350 three times a week for 8 weeks. Terminal blood serum was collected and assayed for pro-inflammatory cytokines, which are upregulated in D2-*mdx* mice. KPT-350 treatment significantly reduced various pro-inflammatory and apoptosis-related cytokines. In addition, KPT-350 treatment significantly increased the expression of anti-inflammatory cytokines that are reduced in vehicle-treated D2-*mdx* mice (J and K). Mean ± SEM. n = 5. ***p < 0.001, ****p < 0.0001, two-way ANOVA with a Tukey correction.

Serum cytokines are a valid biomarker for dystrophin-deficient skeletal muscle in dystrophic mice, dogs, and human patient samples.^54^, ^55^, ^56^ We analyzed whole serum taken from each of the four experimental cohorts via a mouse cytokine array panel to determine whether known biomarkers of dystrophin deficiency were altered in the KPT-350-treated D2-*mdx* mice. Vehicle-treated D2-*mdx* mice showed a significant upregulation of key pro-inflammatory and apoptosis-related cytokines, such as TNF-α, interferon gamma (IFNγ), chemokine (C-X-C motif) ligand 16 (CXCL16), osteopontin (Spp1), IL-1α, IL-1β, IL-2, IL-6, and CD95L, compared with vehicle-treated WT control serum (Figures 5A–5I).^22^, ^55^, ^57^, ^58^, ^59^ Whereas KPT-350 treatment did not induce any changes in WT mice, KPT-350 treatment significantly reduced all of these inflammatory cytokines in D2-*mdx* mice compared with vehicle-treated D2-*mdx* mice (Figures 5A–5I). Moreover, levels of these cytokines were not only reduced, but were restored to WT levels. These cytokines have been established to be pro-inflammatory and detrimental in dystrophic pathology.^22^, ^59^, ^60^ In addition, we measured IL-10 and IL-15 that have been previously reported to ameliorate the severity of muscular dystrophy in *mdx* mice.^61^, ^62^, ^63^ These cytokines were significantly lowered in D2-*mdx* mice compared with WT mice serum (Figures 5J and 5K). However, KPT-350 treatment significantly increased IL-10 and IL-15 serum levels in D2-*mdx* mice (Figures 5J and 5K). These results demonstrate that treatment of dystrophin-deficient mice with oral KPT-350 blocks key inflammatory cytokines known to exacerbate dystrophic pathologies, while inducing anti-inflammatory cytokines such as IL-10.

### KPT-350 Increases Macrophage Populations in D2-*mdx* Skeletal Muscle

**Figure 6:**
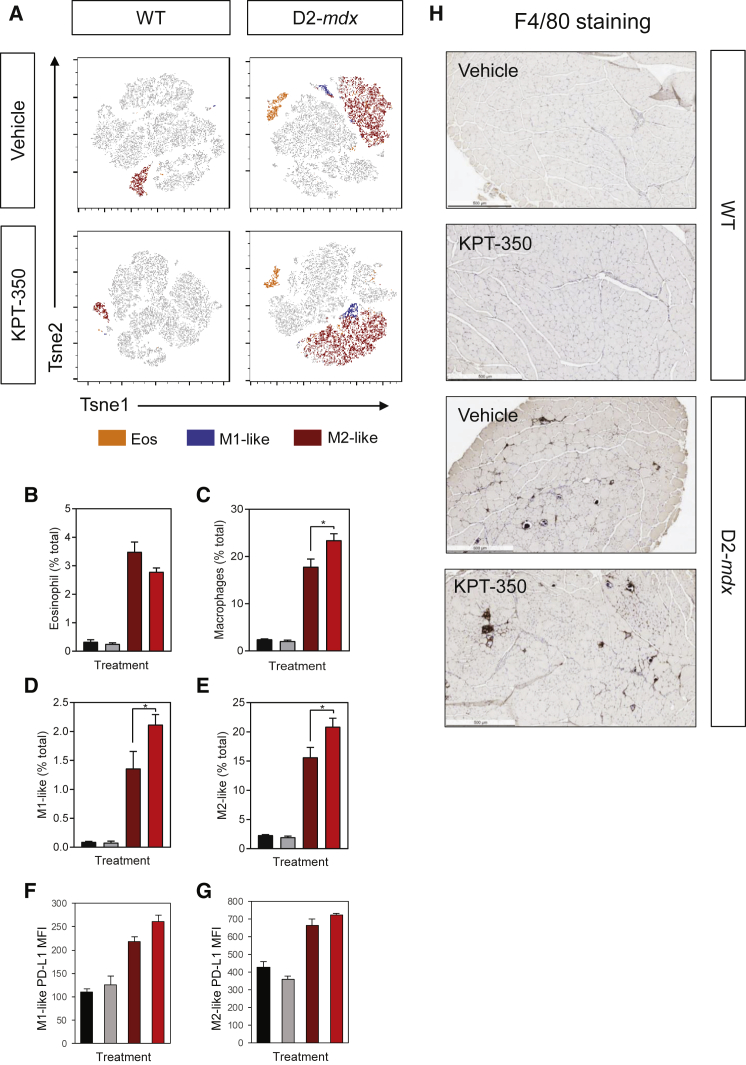
KPT-350 Treatment Increases Overall Macrophage Populations in D2-*mdx* Mice (A) TSNE plots of flow cytometry data gated on live cells of WT and D2-*mdx* mice treated with vehicle or KPT-350. For each color, green represents eosinophils (CD11b^+^F4/80^+^Siglec-F^+^), blue represents M1-like macrophages (CD11b^+^F4/80^+^Siglec-F^−^Ly6c^+^CD206^−^), and red represents M2-like macrophages (CD11b^+^F4/80^+^Siglec-F^−^Ly6c^−^CD206^+^) populations. Eight-week-old WT and D2-*mdx* male mice were given oral KPT-350 three times a week for 8 weeks. Single-cell suspensions were prepared from quadriceps and tibialis anterior muscles for analysis via flow cytometry. (B–E) Quantification of flow cytometry analysis showing the frequency (of total cells) of eosinophils (B), total macrophages (C), M1-like (D), or M2-like (E) macrophages. (F and G) Mean fluorescence intensity (MFI) of PD-L1 in M1-like (F) or M2-like (G) macrophages. (H) F4/80 staining in the tibialis anterior muscle of the four experimental cohorts. Scale bars, 500 μm. Mean ± SEM. n = 5. *p < 0.05, two-way ANOVA with a Tukey correction.

Inflammation-induced muscle damage is a major contributor to the progression and severity of dystrophic pathology. A key component to the inflammatory response is the recruitment of eosinophils and macrophages to damaged muscle. M1-like macrophages are associated with the acute phase of muscle damage. They produce pro-inflammatory cytokines, such as TNF-α, IFNγ, and IL-6, as well as other potentially cytotoxic signals such as nitric oxide (NO) and reactive oxygen species (ROS).^64^ In contrast, M2-like macrophages are involved in muscle repair and regeneration, and inhibit the cytotoxic activity of M1-like macrophages in a IL-10-dependent manner.^64^ Thus, we used flow cytometry to determine the effect of KPT-350 on these immune cell populations in normal and dystrophic muscle. Single-cell suspensions of TA muscles were interrogated by flow cytometry, and t-distributed stochastic neighbor embedding (TSNE) plots were generated to show the distribution of eosinophils, M1-like macrophages, and M2-like macrophages (Figure 6A). KPT-350 treatment did not affect the immune response in WT mice in any parameters measured (Figures 6B–6G). In D2-*mdx* mice, KPT-350-treated mice had significantly higher frequencies of macrophages compared with vehicle-treated D2-*mdx* mice; however, treatment did not have a significant effect on muscle eosinophilia (Figures 6B–6H). Interestingly, programmed death-ligand 1 (PD-L1), a negative costimulatory molecule that suppresses inflammation, was increased on M1-like macrophages, and to a lesser extent on M2-like macrophages isolated from KPT-350-treated versus vehicle-treated D2-*mdx* mice (Figures 6F and 6G). In addition, immunohistochemical staining with anti-F4/80 antibodies of TA muscles demonstrated macrophage infiltration in D2-*mdx* muscles (Figure 6H). Collectively, these data show that despite an overall increase in the proportion of macrophages, KPT-350 promotes a functional reprogramming of muscle inflammation, reflected by increased expression of anti-inflammatory cytokines, PD-L1 expression on macrophages, and decreased expression of pro-inflammatory cytokines.

## Discussion

Our findings indicated that KPT-350 treatment improved muscle histology and serum biomarkers, and blocked overall dystrophic disease progression in DMD zebrafish and mice, and thus further validates the use of SINE compounds to treat DMD symptoms. The lead SINE compound KPT-330/Selinexor is being evaluated in multiple phase 2 and 3 clinical trials in patients with relapsed or refractory hematological and solid tumor malignancies.^65^ These compounds have been shown to block inflammation and reduce neurotoxicity in a rat model of traumatic brain injury (TBI) via regulation of key transcription factors such as FOXP1, FOXO1, and NF-κB.^66^ In a mouse model of Huntington’s disease, KPT-350 treatment reduced nuclear aggregations of protein plaques generated by aberrant transcription and translation of polyQ sequences.^44^ Our findings now demonstrate the therapeutic benefit of the SINE compound, KPT-350, which is known to present the greatest brain penetration among the series of SINE compounds, to block inflammation and overall dystrophic pathologies in DMD zebrafish and mouse models.

In the DMD model of zebrafish, our studies demonstrated the therapeutic efficacy of KPT-350 by the assessment of mutant *sapje* fish and their muscle architecture. KPT-350-treated *sapje* fish had increased muscle integrity and organization as visualized by birefringence and myosin heavy chain (MyHC) staining, respectively. Importantly, long-term dosing with KPT-350 extended the lifespan of the mutant *sapje* fish. These studies provided critical insight into the therapeutic potential of KPT-350 in a DMD animal model.

We next investigated the therapeutic effect of KPT-350 in the mouse DMD model, D2-*mdx* mice. After 8 weeks of treatment, D2-*mdx* mice improved in multiple areas. Histological analyses revealed that KPT-350-treated D2-*mdx* mice had significantly fewer centralized myonuclei and fewer inflammatory areas. Moreover, KPT-350-treated D2-*mdx* showed a higher frequency of large-size fibers with a concomitant decrease in small-size fibers. One potential mechanism for this observation might be that KPT-350 treatment is preventing the degradation of the large type II fibers, which have been shown to be the first to be preferentially degraded in untreated DMD muscle. An alternate explanation is that KPT-350 treatment promotes hypertrophic pathways, because we observed changes in WT muscle in addition to D2-*mdx* muscle. KPT-350 treatment blocks the nuclear export activity of XPO1, which is known to be responsible for the exportation of more than 200 proteins. Some of these are transcription factors, such as FOXO1, and AKT/mechanistic target of rapamycin (mTOR)-related proteins that could influence myofiber metabolism and fiber size.^67^ In addition to improved dystrophic muscle histological markers, we saw an increase in KPT-350-treated D2-*mdx* mice activity compared with vehicle-treated counterparts. Total distance traveled and velocity were increased after KPT-350 treatment, demonstrating that these histological changes are translated into functional changes of the muscle.

In order to elucidate possible mechanisms of KPT-350 actions, we performed a cytokine array for well-known dystrophic cytokines. Most of these cytokines are pro-inflammatory and known to be upregulated in DMD mice and patients.^60^, ^68^, ^69^, ^70^, ^71^ Our findings revealed that although there was no difference in serum levels of any cytokines tested after KPT-350 treatment in WT mice, there were dramatic changes in KPT-350-treated D2-*mdx* mice. KPT-350 treatment of D2-*mdx* mice reduced expression levels of multiple pro-inflammatory cytokines to WT control levels. Importantly, previous studies demonstrated that treating dystrophic mice with neutralizing antibodies for some of these cytokines, such as TNF-α and IL-6, blocks the progression of skeletal muscle degeneration.^22^, ^57^, ^58^, ^60^ Another study demonstrated that ablation of osteopontin improves dystrophic muscle pathologies and promotes muscle regeneration.^55^, ^59^ Our studies are consistent with these reported results because we also found amelioration of muscle disease pathology accompanied by a marked decrease of these cytokines.

Lastly, we investigated the modulation of innate immune responses in dystrophic muscle. KPT-350 treatment increased the proportion of macrophages in D2-*mdx* mice compared with vehicle-treated D2-*mdx* mice. We further analyzed M1-like and M2-like macrophages that are associated with acute pro-inflammatory responses and muscle regeneration, respectively, and found that both populations were increased. Importantly, the potentially detrimental effect of expanded M1-like macrophages is likely negated by their increased expression of PD-L1, which inhibits the activation of immune responses and inflammation. Although it is clear that KPT-350 treatment is altering macrophage activation, the exact functional outcome of this regulation is unknown. Some reports suggest that the balance between M1 and M2 macrophages is tightly regulated through macrophage-intrinsic interactions or extrinsically by regulatory T cells.^7^, ^17^, ^18^, ^72^ These findings, taken together with the reciprocal regulation of pro-inflammatory and anti-inflammatory cytokines and the increased myofiber CSA, suggest that KPT-350 increases the overall regenerative potential of muscle macrophages. This hypothesis is supported by the improved overall histology observed in KPT-350-treated D2-*mdx* skeletal muscles. However, further investigation is warranted into elucidating the specific roles of inflammation and macrophages to fully understand the interplay between dystrophic muscle regeneration and the immune response.

In conclusion, this study demonstrates the novel use of the SINE compound KPT-350 in blocking disease pathology in DMD zebrafish and mouse models. KPT-350 treatment decreased muscle degeneration markers, reduced inflammatory cytokines, and improved overall viability and activity of the animals. Additional experiments into long-term efficacy and systemic effects of SINE compounds in dystrophic mice will be informative toward the advancement of SINE compounds for DMD patients. This study directly contributes to the growing body of knowledge that indicates that reduction of pathologic inflammation and fibrosis can reduce muscle disease symptoms in DMD. Steroid-based anti-inflammatory therapeutics, such as deflazacort and prednisone, have shown therapeutic efficacy in improving muscle strength and are now considered standard-of-care treatment options for DMD patients.^73^, ^74^ Another steroidal compound in clinical trials, vamorolone, has been shown to decrease inflammation while improving symptoms of cardiomyopathy.^75^ With this growing number of therapeutic compounds targeted at preserving muscle function and ambulation, KPT-350 may be a potential candidate considered for pharmacological combinatorial therapy in DMD patients.

## Materials and Methods

### Zebrafish

WT (AB strain) and *sapje* (*dmd*^*ta222a*^ backcrossed onto the AB background over 10 generations) were maintained under standard housing and feeding conditions at the University of Alabama at Birmingham (UAB) Aquatics facility under pathogen-free conditions under the protocol number 20320.^76^ All adult fish were fed a standard diet of *Artemia salina* (brine shrimp) three times per day under a 14-hour on, 10-hour off light cycle in 3-L tanks with a density of no more than 20 fish per tank. WT and *sapje* mutants were genotyped by PCR as previously described.^77^

### KPT-350 Zebrafish Dosing Experiments

For short-term dosing experiments, adult *sapje* heterozygotes were mated and their embryos were collected at 1 dpf. Embryos were then randomly sorted into pools of 25 and placed in six-well plates containing either vehicle (0.01% DMSO/fish water), 0.1 μM KPT-350, 1 μM KPT-350, 5 μM KPT-350, or 5 μM aminophylline (Catalog [Cat.] #A1755; Sigma-Aldrich, St. Louis, MO, USA) dissolved in fish water containing 0.01% DMSO (Cat. #41639; Sigma-Aldrich). A separate initial cohort of 5 μM KPT-350/fish water was found to be toxic to a small percentage of developing fish embryos. For long-term experiments, 4 dpf affected (*sapje*) homozygotes were separated into cohorts of 25 (n = 25 fish per treatment cohort) and given either 0.1 μM KPT-350, 1 μM KPT-350, or 5 μM of aminophylline dissolved in fish water containing 0.01% DMSO. These doses were selected based on comparable doses given for larger juvenile zebrafish evaluated in isolated 1.8-L tanks.^46^ Zebrafish genotypes were confirmed at 21 dpf after tricaine (Cat. #A5040; Sigma-Aldrich) euthanization through Sanger sequencing for the A>T transversion at the zebrafish *dystrophin* exon 4 locus as previously described.^46^ All experiments were performed in a double-blinded (genotype and drug cohorts) fashion to the experimenter, and all experiments were repeated three times (n = 3 independent experimental replicates). All of the KPT-350 drug used in these experiments was synthesized and provided by Karyopharm Therapeutics (Newton, MA, USA).

### Mice

WT (*DBA/2J* strain; stock number 000671) and *mdx* (*DBA/2J* strain; stock number 013141) mice were originally purchased from Jackson Labs (Bar Harbor, ME, USA) and maintained under standard housing and feeding conditions with the UAB Animal Resources Facility under pathogen-free conditions under the animal protocol number 20232. All mice were fed a standard diet of mouse chow (Teklad Global Rodent Diet; 16% protein; Cat. #206S; Envigo, East Millstone, NJ, USA) and had *ad libitum* access to food and water.

#### KPT-350 Mouse Dosing Experiments

Because KPT-350 was formulated to be taken via oral consumption, we used peanut butter pellets as a means of delivery. The peanut butter pellets that contained either vehicle or KPT-350 compound were made in a manner similar to those previously described.^78^, ^79^ Commercial peanut butter (Jif Creamy; The JM Smucker Company, Orrville, OH, USA) was mixed with either vehicle or 5 mg/kg (mouse body weight) KPT-350. The 5 mg/kg dose was determined based on a study demonstrating XPO-1 occupancy by SINE compounds as well as KPT-350 toxicity studies.^39^, ^80^ The peanut butter-drug mix was then frozen in 1-mm^3^ squares in plastic molds (Cat. #106A; Ted Pella, Redding, CA, USA) at −80°C for 4 h prior to dosing. To administer the peanut butter drug pellet, we placed each mouse individually within a red plastic cup (Solo Cup, Lake Forrest, IL, USA) with the bottom removed to observe the mouse and deliver the pellet. A clear plastic lid was placed over the cup to prevent the mouse from escaping. Eight-week-old male mice were used at the start of all dosing experiments. Each mouse was given 15 min to eat the pellet in a contained plastic cylinder with a removed top, and the mice were monitored until the pellet was completely ingested. Peanut butter drug pellets were given to the mice at 8 weeks of age for 8 weeks, three times a week, before physiological testing, final tail vein blood draws, CO_2_ euthanasia, and tissue harvest. All of the KPT-350 drug used in these experiments was synthesized and provided by Karyopharm Therapeutics.

#### Immunofluorescent Stainings

Zebrafish larvae were euthanized by tricaine and decapitated for genotyping of their heads via PCR, and their bodies were placed in glass vials containing in 4% paraformaldehyde (Alfa Aesar, Ward Hill, MA, USA) overnight at 4°C with gentle rocking. After two 5-min washes in 1× PBS (Boston Bio Products, Ashton, MA, USA), the larvae bodies were then incubated with 0.1% Tween 20/1× PBS (Boston Bio Products) for 5 min three times at room temperature with gentle rocking. The larvae bodies were then incubated with 3% BSA (BSA fraction V; RPI, Mount Prospect, IL, USA)/0.1% Tween 20 for 45 min at room temperature with gentle rocking. The larvae bodies were then incubated in primary monoclonal antisera diluted 1:50 (F-59 concentrate; fast MyHC; Developmental Studies Hybridoma Bank, Iowa City, IA, USA) overnight at 4°C with gentle rocking. The following day, the larvae bodies were then washed twice in 0.1% Tween 20 for 5 min each, before being incubated with secondary antisera (goat anti-mouse IgG H+L cross-absorbed Alexa Fluor plus 488 secondary antibody; Thermo Fisher Scientific, Waltham, MA, USA) diluted 1:200 in 3% BSA/0.1% Tween 20 for 45 min at room temperature with gentle rocking. The larvae bodies were then washed three times in 0.1% Tween 20, before being mounted on frosted coverslides (Fisher Scientific) and VECTORSHIELD AntiFade Mounting Media (Vector Laboratories, Burlingame, CA, USA) with coverslips for imaging. Slides were imaged with a Nikon TE2000-U inverted fluorescent microscope (Nikon Instruments, Melville, NY, USA) using OpenLab software version 3.1.5 (Improvision/Perkin Elmer, Waltham, MA, USA). The images were later modified in Adobe Photoshop Creative Cloud version 2018 (Adobe Systems, San Jose, CA, USA) for clarity and resolution.

#### Histochemical Stainings and Analyses

Mouse skeletal muscles were immersed in 10% neutral-buffered formalin (Cat. #HT501128; Sigma-Aldrich, St. Louis, MO, USA) overnight before being embedded in paraffin blocks (Cat. #P3683; Sigma-Aldrich). Paraffin blocks were later transversely sectioned from top to bottom on a Leica RM2125 microtome (Leica Microsystems, Buffalo Grove, IL, USA) at a thickness between 5 and 10 μm following a published protocol.^81^ Slides with sections were stained with either H&E (Sigma-Aldrich) or Masson trichrome (Sigma-Aldrich) following the manufacturer’s guidelines. Slides were then imaged on an Omax Trinocular Metallurgical Microscope (Microscopenet.com, Kitchener, ON, Canada), and images were enhanced for clarity in Adobe Photoshop Creative Cloud version 2018.

#### Mouse Cytokine Array Profiling

The mouse cytokine array (Mouse Cytokine Antibody Array 6) was obtained commercially (RayBiotech, Peachtree Corners, GA, USA) and used on 10 μL of whole serum obtained via cardiac puncture at the conclusion of the experiment. Peripheral blood from each mouse was collected at the time of tissue harvest and was immediately stored at −80°C until use for analysis. For all procedures, the manufacturer’s protocol was followed for analyses. Whole blood was thawed on ice, centrifuged at 1,000 × *g* for 5 min, and diluted 1:1,000 in PBS. Serum supernatant was used for cytokine array incubation. After antibody incubation, spot intensities of cytokine array were assessed using a Bio-Rad (Hercules, CA, USA) ChemiDoc XRS imaging system. For all quantification analyses, the cytokine signal intensities normalized to the WT vehicle control group. Array blots were quantified using the ImageJ (Fiji platform) software.^82^

#### Mouse Activity Tracking

Twenty-four hours prior to experiment termination and tissue harvest, mice were analyzed for overall locomotive activity using the ActiTrack software platform (Harvard Apparatus, Holliston, MA, USA) with isolated individual chambers adapted from a previously described protocol.^83^ Mice were adapted to the room and open-field chambers 1 day prior to activity, and were given a 5-min additional adaptation period prior to activity recording. Mouse activity was recorded for 6 min with no external stimulation. EthoVision XT Version 12 (Noldus, Leesburg, VA, USA) was used to analyze all activity measurements.

#### Immunological Profiling Using Flow Cytometry

Single-cell suspensions from pooled quadriceps and TA muscles were generated as previously described.^84^ The interrogation of live macrophage populations was performed via staining with Zombie NIR viability dye (Cat. #423105; BioLegend, San Diego, CA, USA), CD11b (PerCP-Cy5, Cat. #101228; BioLegend), F4/80 (PE, Cat. #123110; BioLegend), Siglec-F (BV421, Cat# 56268; BD Biosciences, San Jose, CA, USA), Ly6C (FITC, Cat. #128006; BioLegend), CD206 (Alexa Fluor 647, Cat. #141711; BioLegend). Cells were analyzed using a BD FACSAria Fusion flow cytometer (BD Biosciences), and data were analyzed using FlowJo software (FlowJo, version 10; FlowJo, Ashland, OR, USA).

### Statistical Analyses

For the Kaplan-Meier survival plot (Figure 2B), a log rank test was performed to determine statistical significance. For all other graphs, unless otherwise stated, two-way ANOVA with a Tukey honestly significant difference (HSD) correction was used for comparisons among groups and determination of statistical significance, with an *a priori* hypothesis of *p < 0.05, **p < 0.01, ***p < 0.001, and ****p < 0.0001.

### Study Protocol Approvals

All zebrafish studies were carried out under the approval of the UAB Institutional Animal Care and Use Committee under protocol number 20320. All mouse studies were carried out with the approval of the UAB Institutional Animal Care and Use Committee under protocol number 20323. All protocols were submitted and managed by M.S.A.

## Author Contributions

R.M.H., A.L.R., D.E.G., Y.W., J.J.W., J.M.K., S.A.V., H.C., S.G., and M.S.A. performed all experiments and analyzed data findings. H.C., S.G., Y.L., and S.T. provided the KPT-350 compound and vehicle for all experiments. S.A.V., L.M.K., T.v.G., Y.L., S.T., and M.S.A. planned all experiments and interpreted findings. R.M.H., A.L.R., and M.S.A. analyzed all of the data and wrote the manuscript that was submitted by M.S.A.

## Conflicts of Interest

H.C., S.G., Y.L., and S.T. hold equity in Karyopharm Therapeutics (Newton, MA, USA) and their development of SINE compounds for various diseases and disorders. The rights toward development of KPT-350 are currently owned by Biogen, Inc. (Cambridge, MA, USA). Karyopharm Therapeutics and Biogen, Inc. provided the compound and vehicle for all experiments. L.M.K. is a consultant for Pfizer Inc., Summit Corporation PLC, Dyne Therapeutics, and Sarepta Therapeutics for muscle disease drug therapies. All other authors declare no competing interests.
